# Fast free-energy-based neutral set size estimates for the RNA genotype–phenotype map

**DOI:** 10.1098/rsif.2022.0072

**Published:** 2022-06-15

**Authors:** Nora S. Martin, Sebastian E. Ahnert

**Affiliations:** ^1^ Theory of Condensed Matter Group, Cavendish Laboratory, University of Cambridge, JJ Thomson Avenue, Cambridge CB3 0HE, UK; ^2^ Sainsbury Laboratory, University of Cambridge, Bateman Street, Cambridge CB2 1LR, UK; ^3^ Rudolf Peierls Centre for Theoretical Physics, University of Oxford, Parks Road, Oxford OX1 3PU, UK; ^4^ Department of Chemical Engineering and Biotechnology, University of Cambridge, Philippa Fawcett Drive, Cambridge CB3 0AS, UK; ^5^ The Alan Turing Institute, British Library, Euston Road, London NW1 2DB, UK

**Keywords:** RNA secondary structure, sequence–structure map, genotype–phenotype map, neutral set

## Abstract

The genotype–phenotype (GP) map of RNA secondary structure links each RNA sequence to its corresponding secondary structure. Previous research has shown that the large-scale structural properties of GP maps, such as the size of neutral sets in genotype space, can influence evolutionary outcomes. In order to use neutral set sizes, efficient and accurate computational methods are needed to compute them. Here, we propose a new method, which is based on free energy estimates and is much faster than existing sample-based methods. Moreover, this approach can give insight into the reasons behind neutral set size variations, for example, why structures with fewer stacks tend to have larger neutral set sizes. In addition, we generalize neutral set size calculations from the previously studied many-to-one framework, where each sequence folds into a single energetically preferred structure, to a fuller many-to-many framework, where several low-energy structures are included. We find that structures with high neutral sets in one framework also tend to have large neutral sets in the other framework for a range of parameters and thus the choice of GP map does not fundamentally affect which structures have the largest neutral set sizes.

## 1. Introduction

**Figure 1.**
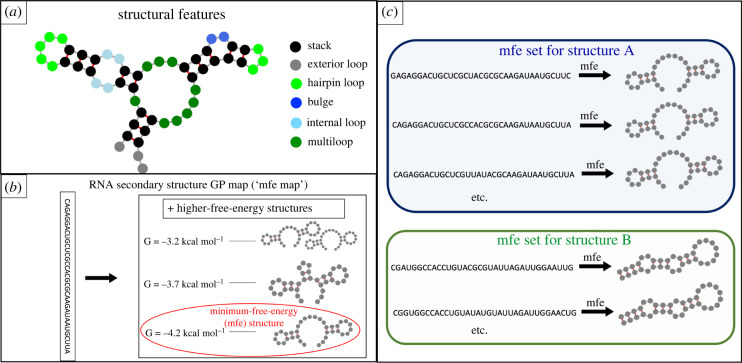
RNA secondary structure folding model and minimum free energy (mfe) set size definition. (*a*) Structural features in a folded RNA molecule: the secondary structure is formed by the formation of base pairs (red lines), with no pseudoknots (i.e. without crossing pairs) [1]. Throughout this paper, we follow the Turner free energy model [2] (using the ViennaRNA [1] implementation) and thus assume that both Watson–Crick and GU ‘wobble’ base pairs can form. A set of adjacent base pairs is called a *stack* (or *stem*). All other regions are *unpaired* or *loop* regions and further classified as hairpin/exterior/bulge/multi/internal loops. This description is standard in the field (see similar schematics in [2,3]), except for minor differences in the naming of loop regions. (*b*) Each RNA sequence can fold into a number of structures and the free energy of folding can be calculated for each of these structures with the Turner [2] free energy model. Here all structures with free energies *G* ≤ −3.2 kcal mol^−1^ are shown for the sequence CAGAGGACUGCUCGCCACGCGCAAGAUAAUGCUUA as an example. The free energy *G* is computed relative to the unfolded structure, i.e. the structure with no base pairs [4]. When computing neutral set sizes, it is usually assumed [5] that each sequence folds only in its minimum-free-energy (mfe) structure (circled) and so there is a unique structure for each sequence (*many-to-one map* [5]). However, in practice several low-energy structures will fold if they have similar free energies, and thus we can build a map, where each sequence folds into several low-energy structures (a *many-to-many map* [5]). (*c*) Schematic for the mfe set: the mfe set of a given structure contains all sequences whose mfe structure matches the given structure (usually referred to as *neutral set*, but here we use the term mfe set to distinguish it from the second neutral set studied in this paper, the low-energy set). Panel (*b*) is adapted from [6].

Functional RNA molecules fold into well-defined structures and perform biological functions. Research on how these structures evolve has focused on the secondary structure, i.e. the pattern of base pairing of folded RNA strands (as illustrated in figure 1*a*).

For the evolution of RNA secondary structures, one factor has been shown to be particularly important [7–9]: the neutral set size of a structure, which quantifies how many sequences fold into that structure [9]. There are several reasons why a high neutral set size can make it more likely for a structure to evolve: for a given structure to fix in a population, it first has to appear in the evolving population through random mutation and structures with high neutral set sizes are more likely to be generated through random mutations [7,9]. Even a structure with a selective advantage is only likely to be acquired if it appears a sufficient number of times and a structure that does not appear even once in the relevant time scale cannot evolve [9]. A second factor favouring structures with high neutral set sizes is that they tend to be more robust to mutations [10,11] and higher robustness can be selected for in populations evolving at high mutation rates since it ensures that mutations are less likely to be deleterious [12]. Thus, models predict that neutral set sizes are crucial for the outcomes of evolutionary processes and this prediction has been supported by data from functional RNA sequences [7,8,11,13]: these data suggest that the functional RNA structures that have evolved in nature are structures with high neutral set sizes [7,8,11,13], even when accounting for thermodynamic stability as a confounding factor [7]. Owing to its relevance for evolutionary processes, several terms exist for the neutral set size and related quantities: it has also been referred to as *phenotype abundance* [7] or occasionally *neutral network size* [11], but both of these terms are ambiguous since some authors [5,14] use them synonymously with neutral component size to describe the number of sequences, which not only correspond to the same structure, but are also connected by a network of neutral mutations.

To analyse the genotype–phenotype (GP) map and compute neutral set sizes, we need a method of obtaining secondary structures from sequences. For this, computational methods are used, which implement the following model: each sequence can fold into a number of structures (schematic in figure 1*b*). A free energy value can be calculated for each of these structures according to the Turner free energy model [2,15,16], as implemented by the ViennaRNA package [1]. The free energy depends on both sequence and structure [15]. A structure with lower free energy is more likely to fold and so a common convention is to assume that each sequence folds solely into its minimum-free-energy (mfe) structure (e.g. [7–9,11,14]).

With this structure prediction method, neutral set sizes for mfe structures, here referred to as *mfe set sizes* *N*_mfe_, can be computed by folding all sequences of a given length into their mfe structures and recording how frequently each structure is found [7], as illustrated in figure 1*c*. However, this is only feasible for short sequences because the number of all sequences of a given length *L* grows exponentially as 4^*L*^. Thus two methods have been developed, which generate a sequence sample according to specific criteria, predict the mfe structures in this sample and infer neutral set sizes from these data [11,17]. These sample-based methods are accurate [8,17] and so we will use the sample-based method by Jörg *et al.* [11], referred to as the NNSE [17], as a reference method in this paper. However, sample-based methods are computationally expensive, with >≈ 103 structure predictions per neutral set required even by the faster method [17].

Because of the computational cost associated with structure prediction, there is a demand for faster sample-free methods which distinguish frequent structures with large mfe set sizes *N*_mfe_ from rarer structures with small mfe set sizes *N*_mfe_ directly from structural features such as stacks and loops (as defined in figure 1*a*). Several approaches address this gap: firstly, the contiguity statistic, which is based on several structural features, including the number of base pairs and the number of stacks [7], secondly the two-versatility model, which only takes the number of base pairs into account [14,18] and thirdly a simple structural indicator, the number of stacks [8]. However, these approaches are contradictory: a higher number of base pairs is positively correlated with the computed mfe set size in one case [7], but negatively in another approach [14,18]. In this paper, we will thus revisit the connection between structural characteristics and neutral set sizes and propose a new method, which is rooted in the free energy model of RNA folding. Previously [6], we have found that stability values are linked to neutral set sizes, suggesting that thermodynamic approaches could be effective for neutral set size prediction. However, the mean stability of a structure had to be estimated through sampling and so a fast sample-free approach for neutral set size prediction has to rely on free-energy calculations instead of stability arguments. Therefore we follow concepts developed for protein models in order to use free-energy calculations for neutral set size predictions: for proteins, England & Shakhnovich [19] estimated mfe set sizes *N*_mfe_ by arguing that the neutral set size for a given structure *A*, *N*_*A*,mfe_, is correlated to the number of sequences which have *A* as a low-energy structure, even if not necessarily as the mfe structure. We will refer to this quantity as the low-energy set size *N*(*G* ≤ *x*). There is some evidence that low-energy set sizes are correlated with mfe set sizes in RNA (electronic supplementary material of [13,20]), but here we will use a different definition of the low-energy set size: we define the low-energy set size *N*_*A*_(*G_A_* ≤ *x*) as the number of sequences which fold into a given structure *A* with a free energy *G*_*A*_ lower than *x*. These sequences do not necessarily have to fold into *A* as their lowest-energy (i.e. mfe) structure; *A* can merely be one of several low-energy structures. This definition was chosen because a simple free-energy inequality, *G*_*A*_ ≤ *x*, determines if a sequence is in the low-energy set of structure *A* and this will allow us to estimate low-energy set sizes based on the free energy model. A central component of this paper focuses on the connection between the mfe set size *N*_*A*,mfe_ and the low-energy set size *N*_*A*_(*G*_*A*_ ≤ *x*) in order to apply our low-energy set size predictions to mfe set sizes.

Thus, several neutral set size definitions are used in this paper with the following terminology: the neutral set size in the GP map from sequences to their mfe structures (the ‘mfe map’) will be referred to as the mfe set size *N*_mfe_. In the mfe map each sequence corresponds to exactly one structure and so it is a many-to-one framework [5]. The GP map from sequences to several low-energy structures on the other hand (the ‘low-energy map’) is a many-to-many framework [5] and neutral set sizes in this map will be referred to as low-energy set sizes *N*(*G* ≤ *x*). This definition has one free parameter, the low-energy cut-off *x*. Estimates of these quantities derived from our free-energy-based calculations or other sample-free methods are denoted by a tilde, i.e. as ${\tilde{N}}_{\mathrm{mfe}}$ and $\tilde{N}(G\leq x)$ respectively.

The low-energy set size, *N*(*G* ≤ *x*), is important in its own right because low-energy structures can be close in energy to the mfe structure [21], or even within the resolution of the energy model [22]. A given sequence can therefore fold into several structures. This is not just an artefact of the thermodynamic model: RNAs fold into several structures [23,24] and this can be relevant for their function [23] and observed in their evolution [25]. If low-energy structures matter in the function and evolution of an RNA molecule, then they should be included in the neutral set size and thus the low-energy set size would be a more appropriate way of quantifying how many sequences correspond to a given structure and how likely a given structure is predicted to evolve.

This paper thus analyses the relationship between mfe set sizes and low-energy set sizes, uses thermodynamic arguments to estimate low-energy set sizes and combines these results into a fast and accurate method for mfe set size estimates. This calculation will draw a direct line from free-energy terms in the folding model to neutral set sizes and thus, in addition to being useful for fast large-scale analyses in the future, it provides insight into why the neutral set sizes of some structures are orders of magnitude higher than those of others. This analysis is performed for sequences of length *L* = 35. We use a folding model without isolated base pairs since this is likely to be more realistic [26,27]. Data for alternative sequence lengths and a folding model with isolated base pairs are presented in the electronic supplementary material (section S3).

The paper is structured as follows. Firstly, we compute and compare low-energy set sizes *N*(*G* ≤ *x*) to the corresponding mfe set sizes *N*_mfe_. Secondly, we estimate low-energy set sizes $\tilde{N}(G\leq x)$ based on the free energy model underlying RNA secondary structure predictions. Finally, we synthesize these concepts to predict mfe set sizes ${\tilde{N}}_{\mathrm{mfe}}$ and compare the accuracy of our predictions to previous methods.

## 2. Results

### 2.1. Low-energy set sizes are correlated with mfe set sizes

#### 2.1.1. Low-energy set sizes

**Figure 2.**
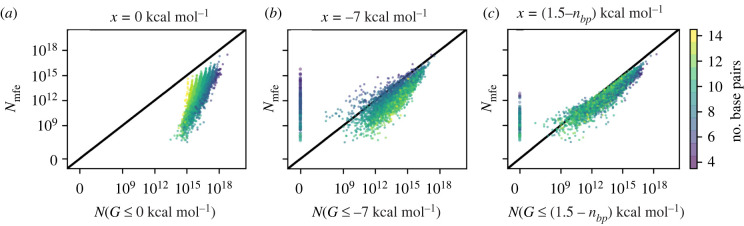
The mfe set sizes *N*_*A*,mfe_ (i.e. number of sequences with *A* as their mfe structure) for a structure sample are plotted against the corresponding low-energy set sizes *N*_*A*_(*G* ≤ *x*) (i.e. number of sequences with *A* as a low-energy structure for which *G*_*A*_ ≤ *x*). The low-energy cut-offs are: (*a*) *x* = 0 kcal mol^−1^ and (*b*) *x* = −7 kcal mol^−1^. Further low-energy cut-offs between −11 kcal mol^−1^ ≤ *x* ≤ 0 kcal mol^−1^ are included in the electronic supplementary material (section S1.3). (*c*) Heuristic: *x* = (1.5 − *n*_*bp*_) kcal mol^−1^, where *n*_*bp*_ is the number of base pairs in structure *A*. The number of base pairs in each structure is indicated by the colour and the black line indicates a one-to-one correspondence (*x* = *y*). A set of 5000 structures of length *L* = 35 is used—these were sampled from a full list of valid structures as described in §5.4. The methods used to compute these set sizes are Jörg *et al.*’s [11] NNSE for mfe set sizes and a custom adaptation for low-energy set sizes, as described in §5.2. This figure is adapted from [6].

First, we will analyse the relationship between the established mfe set size *N*_mfe_ and the low-energy set size *N*(*G* ≤ *x*). The low-energy set size *N*(*G* ≤ *x*) has one free parameter, the low-energy cut-off *x*, up to which structures are included in the many-to-many framework. We will consider several values for this parameter: *x* = 0 kcal mol^−1^ in figure 2*a* and *x* = −7 kcal mol^−1^ in figure 2*b*. Data for a wider range of cut-offs are included in the electronic supplementary material (section S1.3) and support the qualitative trends in figure 2*a*,*b*.

Firstly, we find a linear trend on the log–log scale for both values of *x*. This correlation is strong: the Pearson correlation coefficient between the logarithms of non-zero set sizes is 0.79 for *x* = 0 kcal mol^−1^ and 0.84 for *x* = −7 kcal mol^−1^. Thus, the low-energy set size and the mfe set size are correlated on a logarithmic scale.

Secondly, the low-energy set size *N*(*G* ≤ 0 kcal mol^−1^) in figure 2*a* is greater than the mfe set size, *N*_mfe_, for all structures in the sample. This is because all sequences with a structure *A* as their mfe structure also satisfy the low-energy criterion *G*_*A*_ ≤ 0 kcal mol^−1^ since all mfe structures are lower in free energy than the unfolded structure, which has 0 kcal mol^−1^ by definition. Thus, *N*_*A*_(*G* ≤ 0 kcal mol^−1^) ≥ *N*_*A*,mfe_ is guaranteed to hold for any structure *A*. For the other cut-off (*x* = 7 kcal mol^−1^ in figure 2*b*), no upper or lower limits exist. In fact, structures with a non-zero mfe set size, *N*_mfe_ > 0, can have a zero low-energy set size, *N*(*G* ≤ −7 kcal mol^−1^) = 0. This is true for about 4% of structures in our data. However, this value should be interpreted only as an upper limit on the number of zero low-energy set sizes because it is possible that for some structures, where a small number of sequences with *G* ≤ −7 kcal mol^−1^ exist, these are simply not found with the RNA inverse folding heuristics (as described in §5.2).

Thirdly, the gradients of the linear trends on the log–log scale differ for the two values of *x*: the gradient is greater than one for *x* = 0 kcal mol^−1^, whereas it is close to one for *x* = −7 kcal mol^−1^. Thus, for *N*_mfe_ and *N*(*G* ≤ −7 kcal mol^−1^), the non-zero values span ≈10 orders of magnitude, compared to only ≈5 for *N*(*G* ≤ 0 kcal mol^−1^). In the electronic supplementary material (section S1.1), we model this qualitative difference by using a Gaussian approximation for the free energy distribution of each structure. Essentially, we find that small structure-specific differences in the mean of this distribution have a much bigger effect on the number of sequences which satisfy the stricter free energy criterion *G* ≤ −7 kcal mol^−1^ than the number of sequences which satisfy the more lenient criterion *G* ≤ 0 kcal mol^−1^.

Finally, we consider the number of base pairs, *n*_*bp*_, of each structure, and identify another difference between the data in figure 2*a*,*b*: for a group of structures with a fixed mfe set size, *N*_mfe_ = *a*, *N*(*G* ≤ 0 kcal mol^−1^) is highest for structures with a small *n*_*bp*_, whereas *N*(*G* ≤ −7 kcal mol^−1^) is highest for structures with a large *n*_*bp*_. This observation agrees with previous research, which has argued that the relationship between the structural characteristics and set sizes can be a trade-off between energetics and base pairing constraints [28]. In our case this argument can be applied as follows: on the one hand, base pairs can contribute stacking terms and lower the free energy. Therefore, structures with a high *n*_*bp*_ are more likely to have sequences which satisfy strict free energy criteria like *G* ≤ −7 kcal mol^−1^. On the other hand, structures with a smaller number of base pairs have a higher number of sequences which are at least compatible with the structure [29]. Thus, the number of sequences which meet a lenient free energy criterion like *G* ≤ 0 kcal mol^−1^ is higher for structures with small *n*_*bp*_.

#### 2.1.2. Heuristic approximation of mfe set sizes

This brings us to the key question: if we want to estimate mfe set sizes from low-energy set sizes, which cut-off *x* should we choose? In the following, we will use a simple heuristic: *x*_heuristic_ = (1.5 − *n*_*bp*_) kcal mol^−1^, where *n*_*bp*_ refers to the number of base pairs in a structure. This cut-off was chosen to approximate *N*_mfe_ values with low-energy set sizes, as described in the electronic supplementary material (section S2.1). Figure 2*c* shows that this heuristic approximates *N*_mfe_ well (Pearson correlation *r* = 0.93). Why a different cut-off is used for structures with different numbers of base pairs is discussed in the electronic supplementary material (section S2.3): in brief, we find that the low-energy sequences for structures with a high number of base pairs have an exceptionally high number of competing low-energy structures and so there are many low-energy structures, but only one of them can be the mfe structure. One can imagine that a sequence with high GC content would be one example. To compensate, the heuristic uses a stricter cut-off for structures with a high number of base pairs.

It is important to emphasize that the heuristic is just a method of approximating *N*_mfe_ by arguing that the number of sequences with *G*_*A*_ ≤ *x*_heuristic_ quantifies the energetics of structure *A* is in a way that is relevant for the mfe set size, *N*_*A*,mfe_. However, the heuristic does not suggest that the sequences with *G*_*A*_ ≤ *x*_heuristic_ for a structure *A* will have *A* as its mfe structure. This point is discussed in more detail in the electronic supplementary material (section S2.2). An additional caveat is that about 2.1% of structures are predicted to have *N*_*A*,heuristic_ = 0 even though they have *N*_*A*,mfe_ > 0, but again this may be an artefact of inverse folding heuristics.

#### 2.1.3. Summary: low-energy set sizes and mfe set sizes

To sum up, we found that structures with large mfe set sizes also tend to have large low-energy set sizes for a range of low-energy cut-offs. However, the details of this relationship depend on the cut-off and the number of base pairs in the structure and therefore we constructed a heuristic to link mfe set sizes to low-energy set sizes.

### 2.2. Low-energy set sizes can be estimated without sampling

The link between low-energy set sizes, *N*(*G* ≤ *x*), and mfe set sizes, *N*_mfe_, allows us to estimate mfe set sizes by predicting low-energy set sizes, similar to existing work on neutral set size differences for proteins [19]. Estimating low-energy set sizes from the free energy model is simpler than estimating mfe set sizes: it is easier to determine if *A* is a low-energy structure for sequence *s* than to find out whether *A* is the *lowest*-energy structure of sequence *s*. Thus, we will take the Turner [2] free energy model of RNA folding and estimate, with some simplifications, how many sequences fold into a given structure and satisfy the free energy criterion *G*_*A*_ ≤ *x*.

Since base pairs can only form between certain nucleotides, not all sequences can fold into all structures. Therefore, we can already exclude sequences which cannot fold into *A* at all and thus start our analysis with the *compatible sequences* [30] of *A*, which are the sequences for which *A* is an allowed structure: since the compatibility of a sequence with a structure only depends on whether the sequence has a correct pair of nucleotides at each set of paired positions (either a Watson–Crick or a wobble base pair), the number of compatible sequences can be computed with a simple formula: for a structure with *n*_*bp*_ base pairs, it is given by [29]

2.1 $$N_{C,A}=4^{L-2n_{bp}}\times 6^{n_{bp}}.$$

The fact that a sequence is compatible with a structure is a simple first step, but it says nothing about energetics. Therefore, the key part of the calculation is determining which fraction *f*_*A*_ of compatible sequences meet the low-energy criterion *G*_*A*_ ≤ *x* when folding into *A*.

We will begin with a brief overview of the method: we work with the Turner free energy model [2], but make several simplifications. In this model, the free energy *G*_*A*_ is a sum of free energy terms from loops, stacking interactions and dangling ends/terminal mismatches. Free energy contributions from loop regions are usually positive and thus destabilizing [4]. Stacking terms are usually stabilizing and occur between two directly adjacent base pairs (i.e. in a stack). Dangling ends and terminal mismatches are free energy terms applied to certain loop sites adjacent to base pairs and are also usually stabilizing (as seen from the parameter tables in [2]). The details and parameters for these terms in the Turner free energy model can be found in [2].

We address each of these three free energy contributions in turn to estimate how many sequences fulfil *G*_*A*_ ≤ *x* for a structure *A*. First, we compute the free energy of loop regions *G*_loop_. We simplify this calculation by ignoring sequence-dependent loop free energy contributions. With this approximation, the loop free energy only depends on the structure (details in §5.5.1). Because *G*_loop_ is usually destabilizing, stabilizing free energy contributions from stacks, dangling ends and terminal mismatches are required to stabilize the structure. These terms are sequence-dependent [2] and so only some of the sequences which are compatible with a structure fulfil the low-energy criterion *G*_*A*_ ≤ *x*.

In our treatment of sequence-dependent terms, we will start with stacking terms since they can have higher free energy contributions. The sequence dependence of stacking term is complex and depends on two adjacent base pairs, but on average GU stacking terms are less stabilizing than GC stacking terms (parameter table in [2]). Therefore, depending on energetic constraints, it could be the case that sequences only satisfy the low-energy criterion for *A* if they have GC base pairs at all paired positions. However, it could also be the case that any valid base pair at paired positions is sufficient. This depends on free-energy considerations and many structures are likely to lie somewhere between those two extremes. For these structures, having a GC base pair at a given position will make it more likely for the full sequence to meet the low-energy criterion for *A*, but it is not a strict requirement for there to be GC base pairs at all paired positions for the low-energy requirement to be satisfied. These different levels of constraints on the sequence at individual paired positions can be used to estimate the total number of sequences that satisfy the low-energy criterion for *A* if we describe the different levels of sequence constraints quantitatively: for this, we can use the versatility framework by Manrubia and colleagues [14,18], which we review in more detail in §5.6.1, and quantify the constraints on base pairs by their versatilities. We argue that imposing stricter constraints and restricting base pairs to GC will decrease the versatility and lower the free energy. We assume that this relationship between free energy contribution and site versatilities is a linear one for both base pairs and dangling ends/terminal mismatches and use this approximation to estimate site versatilities. Once we have estimated versatilities based on free-energy arguments, we can use the known relationship between versatilities and neutral set sizes from [14] to calculate low-energy set sizes (details in §§5.5.2–5.6).

**Figure 3.**
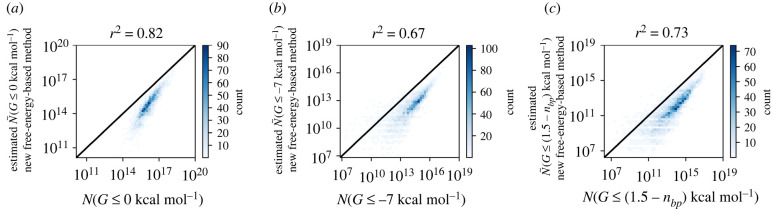
New free-energy-based method for estimating the low-energy set size *N*(*G* ≤ *x*). (*a*) Test of the new method (*y*-axis) against reference data from a custom adaptation of Jörg *et al.*’s [11] NNSE (*x*-axis) for *x* = 0 kcal mol^−1^. (*b*) Similarly for *x* = −7 kcal mol^−1^. (*c*) Similarly for the heuristic: *x* = (1.5 − *n*_*bp*_) kcal mol^−1^, where *n*_*bp*_ is the number of base pairs in the structure. Data for further values of *x* are shown in the electronic supplementary material (figure S5). Tildes are used in the notation to distinguish estimates ($\tilde{N}(G\leq x)$) from the reference values (*N*(*G* ≤ *x*)). We find a high correlation between the two methods on a log–log scale and this is quantified by the coefficient of determination (*r*^2^) shown above the plot. Data for 5000 structures of sequence length *L* = 35 are computed, but structures with *N*(*G* ≤ *x*) = 0 are not included. The black line indicates a perfect correspondence (*x* = *y*). Panels (*a*) and (*b*) are adapted from [6].

Thus, our method of estimating the low-energy set size $\tilde{N}(G\leq x)$ is rooted in the Turner [2] free energy model of RNA folding, but makes a number of approximations, for example, by ignoring sequence-dependent terms in loop regions and by assuming a linear relationship between sequence constraints in stacks and their free energies. To test if our method is accurate despite these simplifications, we plot our low-energy set size predictions against reference data from a low-energy adaptation of an established sample-based prediction method, the NNSE by Jörg *et al.* [11], which relies on the ViennaRNA [1] implementation of the free energy model (figure 3): our predictions are highly correlated with the reference data with a coefficient of determination of *r*^2^ = 0.82 for *x* = 0 kcal mol^−1^, *r*^2^ = 0.67 for *x* = −7 kcal mol^−1^ and *r*^2^ = 0.73 for *x*_heuristic_. However, our estimates systematically underestimate the low-energy set size. One reason may be that we do not perform a full combinatorial calculation of different ways in which a structure may be stabilized, for example, by a combination of dangling end and stacking terms, and thus miss some combinations of stabilizing terms.

### 2.3. Comparison with existing methods for *N*_mfe_ predictions

In the previous sections, we found a heuristic linking low-energy set sizes *N*(*G* ≤ *x*) to mfe set sizes *N*_mfe_ and constructed a method of estimating low-energy set sizes based on free-energy arguments. In this section, we synthesize these results to estimate mfe set sizes: we use the calculations introduced in the previous section to estimate *N*(*G* ≤ *x*_heuristic_), where *x*_heuristic_ = (1.5 − *n*_*bp*_) kcal mol^−1^, and use the result as an approximation to *N*_mfe_.

**Figure 4.**
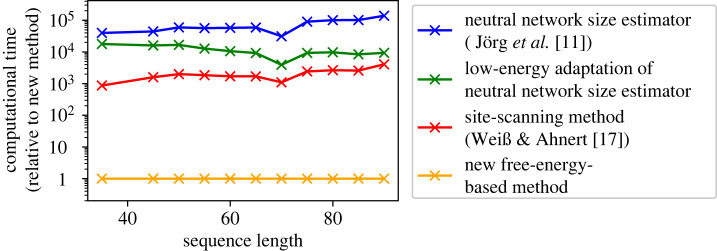
Computational cost of our new method compared to sample-based methods: here, the computational time (relative to the new method) per neutral set size prediction is recorded for structures of different sequence length *L*. Four methods are compared: the NNSE by Jörg *et al.* [11], our custom low-energy set size adaptation of the NNSE, the site-scanning method by Weiß & Ahnert [17] (with a sample size of 1000 and a subsample size of 100) and our new free-energy-based method. The data are based on 30 structures for each sequence length and these are selected randomly from the following datasets: for *L* = 35, the structures from the previous plots were used. For longer sequences, we use sequences in the fRNA dataset compiled by Weiß & Ahnert [17] and apply structure prediction to obtain a structure sample. In absolute terms, the order of magnitude of the runtime for the new method was found to be ≈3 × 10^−3^ s for *L* = 35 and ≈1 × 10^−2^ s for *L* = 75 (on an Intel(R) Xeon(R) E5-2670 processor at 2.30 GHz; more data on absolute values can be found in the electronic supplementary material, figure S29). This figure is adapted from [6].

A key advantage of sample-free methods—including our method—is that they are much more computationally efficient than sample-based methods: our method is about three orders of magnitude faster than existing sample-based methods (figure 4). Our new method is faster even than our custom low-energy set size adaptation of the sample-based approach by Jörg *et al.* [11], which only computes free energies without identifying mfe structures, and is therefore faster than the original approach by Jörg *et al.* [11]. This highlights the difference between sample-based and sample-free approaches: our new method is sample-free and only performs a single free-energy calculation per structure, whereas the low-energy adaptation of the approach by Jörg *et al.* [11] calculates a free energy value for each sequence in a large sequence sample, which contains of the order of approximately 10^5^ sequences [17]. The computational speed of our new method is sufficiently high that we can estimate the mfe set sizes for a full list of all 1.3 × 10^7^ valid secondary structures of length *L* = 35 (data in electronic supplementary material, section S3.3).

To evaluate how accurate these fast predictions are, we will test the accuracy of the mfe set sizes computed with our method against reference data from the NNSE by Jörg *et al.* [11]. Since existing sample-free approaches are also computationally efficient, we will not only test the accuracy of our method, but also compare its accuracy to existing sample-free methods. In our analysis, we include both methods that predict neutral set sizes and methods that only distinguish structures with high *N*_mfe_ from structures with low *N*_mfe_, without predicting the absolute value of mfe set sizes. In order to compare such a range of methods with different objectives, we will focus on how well each method is able to distinguish structures with various *N*_mfe_ values and thus our comparison is based on correlations between predicted and reference values of mfe set sizes, rather than on absolute *N*_mfe_ values. This analysis is performed for the following existing sample-free approaches: the contiguity statistic by Cowperthwaite *et al.* [7], the two-versatility model by Manrubia and colleagues [14,18] and a simple structural indicator, the number of stacks, as suggested by Dingle *et al.* [8]. The two-versatility model was built for neutral component sizes, but García-Martín *et al.* [14] argue that it can be applied to neutral sets for sequences of length *L* > 16. Additional reasons why this is unlikely to matter for our results are discussed in §5.3.

#### 2.3.1. Broad structure sample

**Figure 5.**
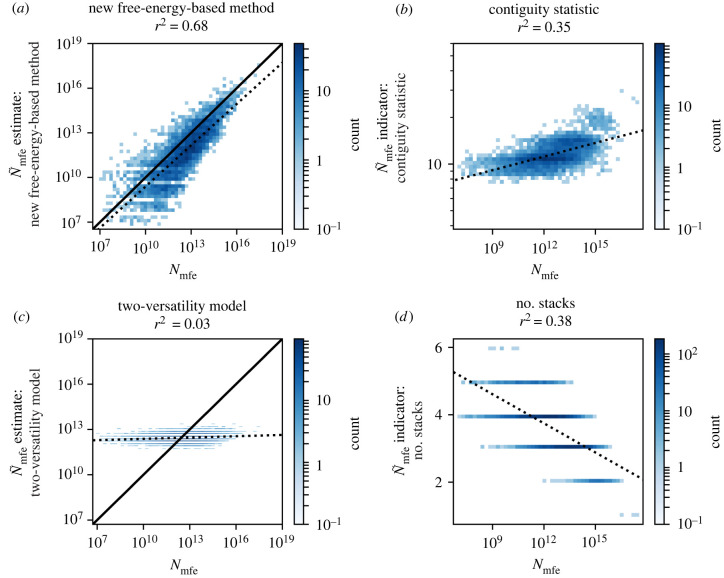
Comparison of the new free-energy-based method and existing sample-free methods for mfe set size ${\tilde{N}}_{\mathrm{mfe}}$ predictions: on the *x*-axis of all four plots, reference *N*_mfe_ values from Jörg *et al.*’s [11] sample-based NNSE are shown. On the *y*-axes, ${\tilde{N}}_{\mathrm{mfe}}$ is estimated with the following fast sample-free methods: (*a*) new method; (*b*) contiguity statistic by Cowperthwaite *et al.* [7]; (*c*) two-versatility model by Manrubia and colleagues [14,18]; (*d*) a simple structural indicator, suggested by Dingle *et al.* [8]: the number of stacks. The *r*^2^ coefficients of a linear fit to the log–log data (drawn as dashed lines) are given above the plots (in *d*, a linear–log plot is used instead). These indicate that our new method performs best, followed by the number of stacks. A one-to-one correspondence (*x* = *y*) is shown as a solid black line in (*a*,*d*) since these two methods compute absolute ${\tilde{N}}_{\mathrm{mfe}}$ values. The other two methods are merely designed to give an indication of which structures are likely to have large *N*_mfe_ values and for this goal, the gradient and sign of the correlation do not matter. Tildes are used in the notation to distinguish sample-free estimates from the reference values. The data are based on 5000 structures of sequence length *L* = 35. This figure is adapted from [6].

First, all four methods are tested on a broad sample of 5000 *L* = 35 structures (figure 5), which is constructed as described in §5.4. Data from the established NNSE method by Jörg *et al.* [11] are used as a reference. We find that our method performs best (*r*^2^ = 0.68 for our method, followed by the structural indicator identified by Dingle *et al.* [8] with *r*^2^ = 0.38).

As discussed above, we cannot compare, how accurately the different methods perform in predicting the correct absolute values of neutral set sizes because two out of the three existing methods only estimate relative neutral set sizes and for the third method, we fitted the parameters on the data itself and not on a separate training set. However, it is still useful to quantify the accuracy of our new method in absolute terms: we find that the RMSD of the $\log_{10}{\tilde{N}}_{\mathrm{mfe}}$ values (as defined in [17]) is ≈1.2. This is consistent with what we observe in figure 5: our method captures the trend and the slope of the reference values, but there is some scatter around this trend and additionally the $\log_{10}{\tilde{N}}_{\mathrm{mfe}}$ values are systematically predicted a little too low, as before for the low-energy set sizes in §2.2. This can be remedied by adding a simple constant offset to the predicted $\log_{10}{\tilde{N}}_{\mathrm{mfe}}$ values. We fit such an offset in the electronic supplementary material (section S3.5.1) and this improves the RMSD to ≈1.0.

#### 2.3.2. Structure samples with specific structural characteristics

**Figure 6.**
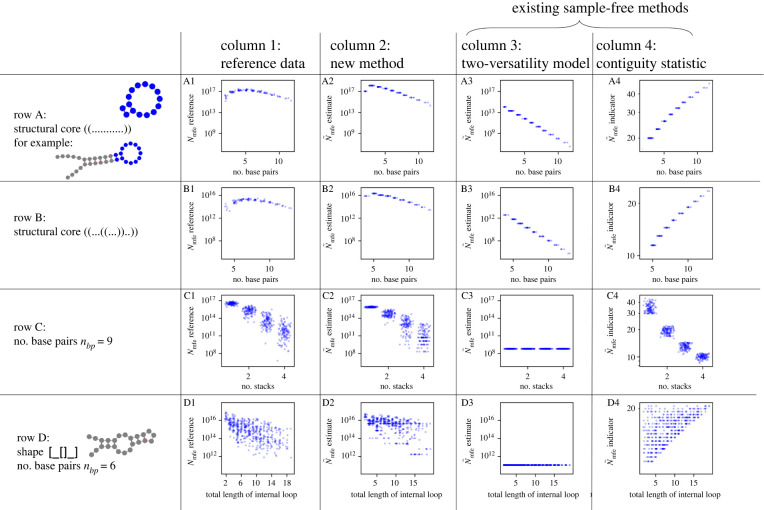
Comparison of our new free-energy-based method against existing methods for specific groups of structures: each row focuses on a specific group of structures and each column shows a different mfe set size prediction method. The columns are: (1) for reference, the prediction with the NNSE method by Jörg *et al.* [11] is shown in the first column. The following columns show sample-free predictions: (2) our new free-energy-based method, (3) two-versatility model by Manrubia & colleagues [14,18] with asymptotic values used for the versatilities, (4) the contiguity statistic by Cowperthwaite *et al.* [7]. The rows are as follows. (A) Dependence of *N*_mfe_ on the number of base pairs for a specific class of structures: all structures have a single stack and a hairpin with exactly eleven unpaired bases: ((...........)) (shown in blue). However, the number of base pairs in the single stack as well as the number of unpaired bases outside the stack (i.e. in the external loop regions at the end of the structure) is varied. The total number of paired and unpaired bases is always chosen such that the length of the structure is constant (*L* = 35). One structure in this sample is shown in grey, with the unchanged part of the structure highlighted in blue. (B) Same principle, but here the unchanged part of the structure is (( ... ((...))..)). Again, the unchanged part of the structure terminates in a stack region. The total number of base pairs in the structure is varied by adding base pairs to this stack and the number of unpaired bases in the external loop regions at the end of the structure can also vary. As before, the overall length of the structure is constant (*L* = 35). The reference data indicate that the impact of the number of base pairs is non-monotonic and accounts for up to ≈2 orders of magnitude in *N*_mfe_ variation. (C) Dependence of *N*_mfe_ on the number of stacks for a sample of structures of constant length (*L* = 35) with a fixed number of nine base pairs (this is the median number of base pairs over all valid structures, as defined in §5.4). The reference data indicate that structures with a higher number of stacks tend to have smaller *N*_mfe_ and that this accounts for around ≈5 orders of magnitude in *N*_mfe_ variation. (D) Dependence of *N*_mfe_ on the total length of internal loop regions for a class of structures of constant total length (*L* = 35) with exactly six base pairs in two stacks, one hairpin loop and one internal loop, and, optionally, exterior loops at the end. This class of structures can be formally described as the level-2 shape [_[]_] in the RNAshapes framework [31], with an additional constraint on the number of base pairs. The reference data indicate that structures with longer internal loop regions have smaller *N*_mfe_. The structure samples in all parts of the plot are derived by filtering the list of all valid structures, as described in §5.4: in rows A and B, all structures which meet the description are included; in row C, a balanced sample was chosen with between 120 and 125 structures per number of stacks; and in row D, 500 structures were chosen from all structures which satisfy the requirement. To reduce overlap of integer *x*-values, Gaussian variation was added, but this variation was limited to ±0.4 to ensure all *x*-values can be read off correctly. Tildes are used in the notation to distinguish sample-free estimates from the reference values from the sample-based NNSE. This figure is adapted from [6].

So far, we have tested our new method on a broad sample of foldable structures of length *L* = 35. Next, we will analyse how the mfe set size depends on structural features (such as numbers of stacks and base pairs) and whether the sample-free prediction methods from above capture these trends. To evaluate the role of structural features systematically, we need to account for the possibility that they are correlated: for example the number of stacks is correlated with the number of base pairs (Pearson correlation coefficient of 0.53 in the sample of structures in figure 5) because each stack consists of two or more adjacent base pairs. Therefore we will analyse groups of highly similar structures, which differ only in as few features as possible to minimize confounding factors (figure 6).

First, the number of base pairs was varied, while the number of stacks was kept constant (figure 6, rows A and B). In addition to keeping the number of stacks constant, we fixed the type and length of as many other structural elements as possible—other than the number of base pairs in a specific stack, only the exterior loops were permitted to vary in length. We find that the number of base pairs is linked to mfe set sizes with a variation of about two orders of magnitude, but this influence is not monotonic. For example in figure 6A1, the neutral set size increases with the number of base pairs for small numbers of base pairs, but decreases for high numbers of base pairs, with a maximum mfe set size *N*_mfe_ at around four base pairs. The same qualitative behaviour is observed in figure 6B1, but with a maximum at around six base pairs. This non-monotonic behaviour is only predicted by our method.

Next, the number of base pairs was kept constant to study variations in the number of stacks (figure 6, row C). We find that a higher number of stacks is linked to lower neutral set sizes, in agreement with [8]. The neutral set size differences observed here are greater than those associated with base pair differences: while structures with one stack have a median mfe set size of ≈4 × 10^16^, structures with four stacks only have a median mfe set size of ≈5 × 10^11^. It is notable that neutral set size differences of this magnitude are found in this sample, where all structures have the same number of base pairs and thus the same number of compatible sequences (according to equation (2.1)). The negative correlation is predicted correctly by our method and the contiguity statistic.

Despite the strong link between the number of stacks and mfe set sizes, there is still a range of *N*_mfe_ values for structures with a constant number of base pairs and stacks in figure 6C1. This indicates that loop types and lengths also play a role and thus our next example focuses on loop length differences within a group of structures which consist of a hairpin loop, an interior loop and constant numbers of base pairs and stacks. We find that there is a weak anti-correlation between *N*_mfe_ and the combined length of the two unpaired regions in the interior loop (figure 6D1). Our method is the only method to predict this weak trend. Again, there is a high variance around the trend, indicating that the details of the other loop regions also play a role.

To sum up, our new free-energy-based method predicts trends across a variety of structure samples. This is confirmed when we plot the sample-free predictions in columns 2–4 against the reference prediction in column 1 (plots shown in the electronic supplementary material, figure S10): for each of the structural samples, our method has the highest correlation with the reference data. It is important to note that the trends observed in this section are not directly built into our method—they emerge from our approximation of the energetics of folding. An additional point to take away from this section is that mfe set sizes can depend on a number of structural characteristics and not all relationships are monotonic. Moreover, the data in figure 6 emphasize that the accuracy of different methods depends on the structural sample of interest. Thus, it is important to test new methods on several test sets of structures. Therefore, the comparison between our new method and existing sample-free methods is repeated for different sequence lengths and structure samples in the electronic supplementary material (section S3.5): in particular, biological fRNA data constitute a typical application for neutral set size calculations (e.g. [7,8,11,13]) and so we use a dataset of fRNA sequences, which was compiled by Weiß & Ahnert [17] and is based on the fRNA database [32].

## 3. Discussion

### 3.1. Structural features and neutral set size

We found the number of stacks to be the structural characteristic that is the strongest single indicator of neutral set size, in agreement with Dingle *et al.* [8]. Our thermodynamic treatment reveals why this might be the case. Firstly, a higher number of stacks means that the structure has a high number of short loop regions between stacks, which tends to be energetically less favourable than arranging the same number of unpaired sites in a small number of long loops (data in the electronic supplementary material, section S3.2). This argument agrees with work on proteins [33], where energetically unfavourable features were shown to have a large impact on neutral set sizes. Secondly, base pairs most effectively stabilize a structure if they are arranged in long stacks, where a higher number of stacking interactions are formed for the same number of base pairs since stacking interactions are only formed between directly adjacent base pairs.

In addition, our analysis of the non-monotonic relationship between the number of base pairs and neutral set sizes agrees with Fontana’s [28] description of a trade-off between compatible sequences and energetics. For the number of base pairs, we find the following trade-off: on the one hand, a higher number of base pairs means that the number of compatible sequences is lower (as seen from equation (2.1)); on the other hand, a higher number of base pairs can provide stabilizing free energy contributions and enable a higher fraction of compatible sequences to fold into the required structure with low energy. Our work allows us to model this non-monotonic relationship quantitatively.

### 3.2. Choosing a genotype–phenotype map definition

Since the minimum-free-energy criterion is not always unique [22] and suboptimal structures are often close in free energy to the minimum-free-energy structure [21], several low-energy structures per sequence can be relevant functionally [23] and in evolutionary processes [24,25]. Therefore, realistic models of the RNA GP map should not restrict themselves to the mfe structure for each sequence, but more complex many-to-many models should be used, which include several low-energy structures for each sequence. Computing the low-energy set size instead of the mfe set size is a key step towards understanding such a many-to-many treatment and therefore the low-energy set size is interesting in its own right.

As soon as we move beyond the mfe set size, we have a free parameter that determines where the line between the relevant low-energy structures and the remaining higher-energy structures is drawn. In our analysis, this is the free energy cut-off *x* and this parameter needs to be chosen depending on the context: if a given molecule is able to perform its function even when it is merely one of many low-energy structures, then the low-energy set size with a lenient cut-off *x* would be appropriate. If, however, a structure needs to be the mfe structure or have a certain Boltzmann frequency in order to function correctly, then the mfe set size or other models would be appropriate.

One key finding from our analysis is that the choices made in selecting a GP map model and setting the cut-off *x* have a much smaller impact than might be expected: we found that mfe set sizes and low-energy set sizes with different values of *x* are correlated, in agreement with previous work on different low-energy set size definitions (electronic supplementary material of [13,20]). Therefore qualitative results, for example which structures have the largest neutral sets, do not depend strongly on the exact definition of the GP map, but instead hold for both the mfe map and the low-energy map for a range of *x*. However, some quantitative differences exist: for example, the *phenotypic bias*, i.e. the difference in neutral set size between frequent and rare structures [9], differs for different definitions of the low-energy set size (as seen from the different gradients in figure 2).

### 3.3. Correlations and prediction of absolute *N*_mfe_ values

In this paper, we followed Cowperthwaite *et al.* [7] and evaluated the quality of our predictions using correlations rather than absolute values. This is sufficient for most applications: for example, research on proteins often relies on contact map traces and contact densities [34–36] as a proxy for neutral set size, even though this does not give absolute neutral set size values.

However, if absolute values were important, figure 5 suggests that even a single constant offset parameter *b* could improve the agreement between the predicted $\log_{10}{\tilde{N}}_{\mathrm{mfe}}$ data and the reference log_10_*N*_mfe_ data. In general, the best choice of the parameter *b* would be likely to depend on the sequence length *L*, similar to the correction parameter in a recent sample-based method, the site-scanning method [17] and the parameters in a recent sample-free method, the two-versatility model [14]. We introduce such an offset parameter *b* in the electronic supplementary material (section S3.5.1), and find that a good choice is *b* ≈ 0.05 × *L* − 0.6. When testing this offset on sequence lengths that were not used to fit *b*, we find that it improves the accuracy of the predicted $\log_{10}{\tilde{N}}_{\mathrm{mfe}}$ values: with the correction, the neutral set sizes of structures of *L* = 100 can be predicted to within ≈1.5 orders of magnitude (≈1.0 order of magnitude for *L* = 35 and *L* = 50, ≈1.2 orders of magnitude for *L* = 70 and ≈1.5 orders of magnitude for *L* = 100). For L >≈⪆ 50, this accuracy is close to that of a recent sample-based method [17], but our free-energy-based method is about three orders of magnitude faster, as shown in figure 4.

More sophisticated fits might further improve the agreement between the predicted $\log_{10}{\tilde{N}}_{\mathrm{mfe}}$ data and the reference log_10_ *N*_mfe_ data. However, for us to confidently fit more sophisticated models for sequence lengths longer than *L* = 35, we would need a better reference dataset of structures and their neutral set sizes: currently, we rely on a dataset of functional RNA sequences (originally from the fRNA database [32], but here the compiled data from [17] are used). These data are shown in the electronic supplementary material (section S3.5). The advantage of the dataset is that fRNA structures are a likely application of our new model. The disadvantage, however, is that fRNA structures make up a biased dataset since they have been found to be predominantly structures with large neutral set sizes [7,8,11]. For this paper, we have generated a broader dataset for intermediate-length sequences of length *L* = 35 as well as datasets with specific conditions (for example, a constant number of stacks) and relied on this dataset throughout our analysis. However, even this dataset may be biased due to its reliance on inverse folding heuristics (as discussed in §5.4) and our approach is only feasible for short sequence lengths since it relies on a full list of valid structures. Future work should design methods to obtain such datasets, both broad samples and samples with specific characteristics, for longer sequences, for example by building on methods in [37]. These would be useful for benchmarking neutral set size prediction methods, but also as null models, against which the neutral set size or robustness of functional RNA structures could be compared.

### 3.4. Designability

One caveat of our method is that, like other sample-free approaches, our method is not suitable for filtering out undesignable structures, i.e. structures which are not the mfe structure for a single sequence. Whether a given structure is undesignable is itself a complex theoretical problem [38]. However, in practice, this should not matter when applying this algorithm to biological fRNA structures, for which it is already known that they are ‘designable’.

## 4. Conclusion

In this paper, we focused on two neutral set size definitions: firstly the mfe set size *N*_mfe_ (i.e. the number of sequences for which the structure is the mfe structure) and secondly the low-energy set size *N*(*G* ≤ *x*) (i.e. the number of sequences which fold into a structure with low energy, but not necessarily as the mfe structure). We found that low-energy set sizes vary over several orders of magnitude, just like mfe set sizes, and that the logarithms of low-energy set sizes and mfe set sizes are correlated. The gradient of this correlation and the number of outliers was found to depend on the choice of low-energy cut-off, *x*.

We then used this link between mfe set sizes and low-energy set sizes to estimate mfe set sizes: we studied the free energy model of RNA secondary structure folding with a number of approximations and computed how many sequences would meet the low-energy criterion *G* ≤ *x* for a given structure. This enabled us to make mfe set size and low-energy set size predictions analytically, without the computationally expensive step of running structure predictions on large sequence samples. We compared our predictions, as well as those of previous sample-free determinants of neutral set size [7,8,14,18], to reference data from a computationally expensive, but accurate sample-based method by Jörg *et al.* [11]. Our new method outperformed existing sample-free approaches. A Python implementation of this method is available (https://github.com/noramartin/free_energy_based_neutral_set_size_prediction).

These results are based on computational structure predictions from the ViennaRNA package [1] and its underlying free energy model [2,15,16]. However, our work would have value beyond the current free energy parameter set because our focus on the free energy model itself means that free energy parameters could easily be updated in our method if improved parameter values were released. One interesting direction for future research could be to extend this method to structures with some tertiary contacts, for example, pseudoknots for which free-energy-based folding methods also exist [39].

An additional caveat is that, like previous sample-free methods [7,8,14,18], our method simply assumes that input structures have non-empty neutral sets. Whether this assumption holds for a particular structure has to be tested using existing methods for inverse folding, such as those described in [1,40,41].

Future research should apply our fast neutral set size estimates to biological data, such as the huge number of fRNA structures available in the RNAcentral [42] database. This could follow similar ideas as in protein research, where the discovery of a simple structural determinant of neutral set size [19] has led to a range of research on neutral set sizes of biological sequences and structures [34–36]. Since the mutational robustness of a structure is correlated with the logarithm of its neutral set size [10,11], the new method will allow us to compare not only the neutral set size, but also the robustness of structures in biological databases. The computational speed-up achieved through this method compared to sample-based methods will be especially useful to improve the accuracy of null models, for example when comparing the neutral set sizes of fRNA structures to the neutral set size distribution of a whole class of structures, whether these are structures formed by sequences of comparable composition (as in [7]) or whether the full neutral set size distribution of all structures is required (as in [8]).

Moreover, future work could investigate the low-energy sets in more detail: in the low-energy map, each sequence can correspond to several structures and therefore belong to several low-energy sets (the neutral set of each of the structures). Finding out whether certain neutral sets share larger overlaps than others would be important for evolutionary processes for which some structures have a selective advantage and others are deleterious.

An additional question for further research is how neutral set sizes in the many-to-one map are related to those in the corresponding many-to-many map in other models: for example the Polyomino model for protein quaternary structure can be treated either in a many-to-one [43] or in a many-to-many [44] framework, depending on how non-deterministic cases are handled.

## 5. Methods

### 5.1. Structure predictions

Structure predictions and inverse folding were performed with the Python bindings of the ViennaRNA package [1,15,16,21,45] (version 2.4.14). Default parameters were used except for the parameter for isolated base pairs: they were not permitted because they cannot form stabilizing stacks in the Turner [2] model and are thus thermodynamically not very stable [26]. However, data for structure predictions with isolated base pairs are included in the electronic supplementary material (section S3.6). Secondary structure illustrations were created using the forna tool [46].

The choice of sequence length is always a compromise between shorter sequence lengths, for which structure predictions are faster, and longer sequence lengths, for which a greater range of structures can fold [47]. Here we use sequences of length *L* = 35, which is long enough for a complex range of structural possibilities to exist, as seen in [26]. Data for alternative sequence lengths are presented in the electronic supplementary material (section S3).

### 5.2. Reference method for *N*_mfe_ and *N*(*G* ≤ *x*) predictions

To test our new method and analyse neutral set sizes, we rely on an established prediction method: the neutral network size estimator (referred to in [17] as NNSE) for mfe set sizes by Jörg *et al.* [11]. This program is accurate [8] and two existing prediction methods were calibrated against data from the NNSE, either directly [17] or to *N*_mfe_ distributions (in [14], based on NNSE calculations from [8]). We changed the setting for isolated base pairs, so that sequences are folded without isolated base pairs.

For *N*(*G* ≤ *x*) predictions, we wrote a custom adaptation of the NNSE algorithm and tested this method against sequence-sampling (electronic supplementary material, section S6). The following adaptations were made compared to the original NNSE algorithm:

1. *Inverse folding*: at the start of the algorithm, a sequence *s* is required which meets the low-energy criterion *G*_*A*_ ≤ *x* for structure *A*. For a strict free-energy criterion (such as *G*_*A*_ ≤ −5 kcal mol^−1^), not every sequence *s* with mfe structure *A* will satisfy this criterion and therefore ViennaRNA’s inverse folding program cannot be used. We thus start with the following three educated guesses: firstly, we use a sequence with GC base pairs at all paired positions and base A at all loop positions; secondly, we run ViennaRNA’s [1] inverse folding program; and finally, we use the initialization step of the INFO-RNA program [40]. If one of these sequences fulfils the low-energy criterion, it is used as a start sequence. If not, we choose the lowest-energy sequence out of the three options and perform a random walk in sequence space with up to 5000 point mutations and base pair swaps to decrease *G*, thus using a similar approach to the local optimization in ViennaRNA’s inverse folding [45]. This inverse folding method is likely to be biased, just like inverse folding methods for the mfe criterion are known to be biased [48], but the NNSE method is not sensitive to bias in the inverse folding method [11]. However, there is no guarantee that our heuristic will converge, even if a solution exists. This is also the case for inverse folding methods for the mfe framework [41].
2. *Distance metric*: the algorithm requires that each sequence can be sorted into one of several nested sets, depending on how similar its folded structure is to the structure of interest *A* [11]. In our case, the GP map is many-to-many and so we use a slightly different distance metric, which only depends on the free energy of the structure of interest: *d* = max(*G*_*A*_ − *x*, 0 kcal mol^−1^). This distance *d* is zero if the low-energy criterion *G*_*A*_ ≤ *x* is satisfied for the given sequence *s*. We bin the distance to obtain 11 nested sets: *d* = 0 kcal mol^−1^, *d* ≤ 1 kcal mol^−1^, *d* ≤ 2 kcal mol^−1^, … , *d* ≤ 9 kcal mol^−1^ and one set with no upper limit.

For both versions of the NNSE, the following parameters were used: three measurements, 2000 initialization steps and 2000 measurement steps (i.e. the default values except for the number of measurements, for which lower values save computational costs while maintaining high accuracy [8]). For sequence lengths *L* > 40 used in the electronic supplementary material (sections S3.5 and S3.6), the number of measurements is set to two. If no convergence is reached, we repeat the analysis with a tenfold greater number of measurement steps.

### 5.3. Implementing existing sample-free methods

The contiguity statistic by Cowperthwaite *et al.* [7] was implemented following the schematics in [7,49]. We assume that multi-loops are treated like exterior loops in the calculation of ‘stem-loop lengths’.

For the two-versatility model by Manrubia and colleagues [14,18], two parameters are needed: the versatility at paired and unpaired sites. A fit is used for the dataset in figure 5. Asymptotic values from [14] are used in figure 6. The two-versatility model was technically developed to compute neutral component sizes (referred to as ‘abundances’), but the authors argue that for sufficiently long sequences of *L* > 16 (i.e. the lengths considered in this paper), the distinction between neutral components and neutral sets becomes irrelevant and thus they fit their model against neutral set size data [14]. Weiß & Ahnert [17] have disagreed with this view and argued that to convert from neutral component sizes to neutral set sizes, a factor of $2^{n_{bp}}$ is required, where *n*_*bp*_ is the number of base pairs in a given structure.

It is easy to show that neither the correction factor nor the chosen versatility parameters affect the results if the agreement between the predictions and the log_10_ *N*_mfe_ data are evaluated using *r*^2^ coefficients, as is the case in this paper: the versatility model from [18] gives $\log_{10}{\tilde{N}}_{\mathrm{mfe}}=a\times n_{bp}+b$, where *a* and *b* are constants that merely depend on the versatility parameters, the sequence length and whether the correction factor for neutral components is applied. Thus, on a logarithmic scale, changing the model parameters simply amounts to a linear transformation of the predicted mfe set sizes and this would not affect the computed *r*^2^ values.

### 5.4. Structure sample

A valid secondary structure has a matching closing bracket for each opening bracket, hairpin loops have a minimum length of three bases and there are no pseudo-knots [1]. Applying these requirements allows us to generate all >1.3 × 10^7^ valid structures of length *L* = 35, following our previous work [50]: essentially, we start with a list of starting symbols (either a dot or an opening bracket) and extend these recursively. At each step, we append each of the three dot–bracket symbols (dot, opening bracket and closing bracket), unless adding a certain symbol would make it impossible to turn the string into a valid structure of length *L* = 35 (for example, by opening more brackets than could be closed, closing more brackets than have been opened, creating a hairpin loop below the minimum length, etc.).

The total number of valid structures is too high to include all structures in this analysis and thus we work with samples of structures: we shuffle the list of all structures and choose the first *n* structures. We only consider structures for which a sequence can be found after 10 iterations of the inverse folding program RNAinverse [45] since we are not interested in structures which may not have a single sequence folding into them as their mfe structure (‘undesignable’ structures [37]). This method was used to generate the structure sample of 5000 structures of length *L* = 35, which is used in figures 2–5. Structure samples with special characteristics (e.g. figure 6) can be generated from the full list of structures in the same way—the list of full structures is simply filtered for structures with the required characteristics and then this filtered list constitutes the starting point for the structure sample.

It is important to note that this sample may not be an unbiased sample of all foldable structures: some structures are falsely discarded as undesignable and thus excluded from the sample, just because the RNAinverse program fails to converge. Here we used 10 iterations of the RNAinverse program as a trade-off between computational feasibility and accuracy: for a sample of 100 valid secondary structures, we found that inverse folding was successful after the first run for only 19 structures, but by the tenth run, 41 structures had a successful inverse folding. The number of successful inverse folding runs gradually increased further to 63 after the thousandth run, but in the trade-off between computational feasibility and performance, we chose 10 repetitions. It is important to note that 10 repetitions are already much better than a single repetition since the number of structures with successful inverse folding had doubled in that interval in our example. However, despite this, there are some structures that are falsely discarded as non-folding and it is likely that out of all designable structures, there are some structures for which RNAinverse is particularly unlikely to converge, presumably structures with low neutral set sizes. These structures would be under-represented in our final sample. To investigate the impact of this potential issue, we generated a smaller sample of 100 structures, where we did not discard structures after 10 attempts, but ran RNAinverse up to 100 times—our method still outperforms existing methods on this sample, albeit with a lower *r*^2^ than before (electronic supplementary material, figure S12). In addition, we worked with structures of sequence length *L* = 13, where it feasible to fold all sequences without relying on inverse folding at all, and found the same (electronic supplementary material, figure S15). Finally, we note that since most structures have lower *N*_mfe_ values than random sequences [8], a small bias towards falsely discarding some of these structures is likely to still leave some low-*N*_mfe_ structures in the sample.

For sequence lengths of *L* > 40 (in figure 4 and the electronic supplementary material, sections S2.4, S3.5 and S3.6), it is not feasible to generate a full list of valid secondary structures and therefore we use a different method: our structure samples rely on non-coding RNA sequences compiled by Weiß & Ahnert [17], based on the fRNA database [32]. We simply take the predicted mfe structures for these sequences (unlike in [17], we compute these without isolated base pairs) as our structure sample.

### 5.5. Structural characteristics for the free-energy-based calculations

In this section, we describe how we calculate the quantities *G*_loop_, *n*_*bp*_, *n*_stack_ and *n*_eos_, which will be used in our free-energy-based neutral set size estimation in the next section (§5.6). The approximations to the Turner [2] free energy model made in this and the following section are only used in our free-energy-based *N*(*G* ≤ *x*) estimates, but not in the reference data, which relies on sample-based methods using the ViennaRNA [1] implementation of the Turner [2] free energy model.

#### 5.5.1. Loop free energy calculation

To estimate *N*(*G* ≤ *x*), our first step is to compute the free energy contribution of all loop regions of a structure, *G*_loop_. Dangling end and terminal mismatch contributions are not included in our loop free energy calculation, since these are considered in a later step. In the Turner [2] free energy model, loop free energy terms depend on both sequence and structure. For the feasibility of our subsequent calculations, we need a single *G*_loop_ value per structure and therefore do not consider sequence-dependent terms. This is likely to be a good approximation for most sequences because most sequence-dependent terms only apply in special cases (for example, internal loops where both unpaired regions consist of a single G base [15]). We test in the electronic supplementary material (section S3.1) whether these approximations mean that our predictions become less accurate for structures with a higher number of bulges, internal loops and multi-loops and find that, while the total loop length has a minor systematic bias, the number of loop regions does not play a role for the accuracy of our method.

With these assumptions, *G*_loop_ is simply the sum of all initiation free energies and the asymmetry contributions for internal loops. Both of these terms depend on the loop types and lengths and can be computed for a given structure following [2,15,16]. We round all free energy contributions to 0.1 kcal mol^−1^.

#### 5.5.2. Number of stacking terms

The dominant stabilizing free energy contribution in RNA is the stacking of base pairs (*bps*). Coaxial stacks are not included in the default settings of ViennaRNA [51] and thus not considered here. Thus, stacking terms can be found only between two contiguous base pairs and across a length-one bulge [2,15]. Thus, the number of stacking terms *n*_stack_ can be read off from the structure—note that this usually differs from the number of base pairs *n*_*bp*_ since the Turner [2] free energy model does not include free energy terms for base pairs themselves, but instead the free energy terms in paired regions depend on the stacking interactions between base pairs.

#### 5.5.3. Number of dangling ends and terminal mismatches

Dangling ends and terminal mismatches also provide free energy terms in the Turner [2] model, which are mostly stabilizing [2]. The default settings of ViennaRNA apply a heuristic to these terms: the program does not test if a loop site is counted twice for the dangling ends of two helices [52]. Thus we also choose a simplified treatment: we treat dangling ends and terminal mismatches as one single type of term, which we will call end-of-stack term, and count the number of end-of-stack-terms, *n*_eos_, as follows:

— one end-of-stack-term per stack end with an adjacent hairpin loop of length four or more,
— one end-of-stack-term per stack end with an adjacent internal loop with at least two loop sites on each strand and at least three sites in the longer strand, and
— one end-of-stack-term for each stack end with an adjacent multi-loop or exterior loop.

These criteria approximately follow the treatment of dangling ends and terminal mismatches in the Turner [2] free energy model.

### 5.6. Free-energy-based *N*(*G* ≤ *x*) estimates

Once the loop free energy *G*_loop_ is computed (as described in §5.5.1), we estimate how many sequences meet the free energy criterion *G* ≤ *x*. These sequences need to offset the positive loop free energy *G*_loop_ with stabilizing contributions from stacking terms and, to a smaller extent, end-of-stack terms. In our simplified treatment, we assume that the sequence-dependent energy is provided primarily by stacking terms since stacking free energies can have higher absolute values. Thus, we assume as a first approximation that each end-of-stack term simply contributes at least *a* = 0.5 kcal mol^−1^, independent of the sequence (this is a good assumption for terminal mismatches, where 99% of values in the corresponding parameter table in [2] are at least as stabilizing as *a*; for dangling ends, less stabilizing values are more common, but *a* is still of the typical order of magnitude of a dangling end contribution in [2]). Thus, we can write the low-energy criterion as

5.1 $$G_{\mathrm{seq}-\mathrm{dependent}}\leq x-(G_{\mathrm{loop}}-a\times n_{\mathrm{eos}})$$

and

5.2 $$G_{\mathrm{seq}-\mathrm{dependent}}\leq G_{\max}.$$

Here the terms on the right have no sequence dependence and thus we will summarize these as *G*_max_ = *x* − (*G*_loop_ − *a* × *n*_eos_). This *G*_max_ is usually negative. The terms on the left are the sequence-dependent contributions for stacking and end-of-stack terms. These sequence-dependent contributions are the reason why only some of all compatible sequences satisfy the low-energy criterion in equation (5.2). To estimate the number of such sequences, we will use a versatility approach. Before we go through this calculation, we will give some background on versatility approaches from [14,17,18,53].

#### 5.6.1. Review of versatility framework

The basic idea behind the versatility approach and related approaches in [14,17,18,53] is that we can estimate neutral set sizes, or neutral component sizes (depending on the chosen model), by focusing on each sequence position individually: for example, if a given structure *A* is only likely to be the mfe structure if there is a U at sequence position 1, either a C or G at sequence position 2 and any letter at position 3, then we can calculate how many sequences satisfy these requirements: 1/4 letters are permitted at position 1 and 2/4 at position 2 and 4/4 at position 3, so, in total, out of the 4^3^ sequences of length *L* = 3, the number sequences fulfilling all three requirements is

5.3 $$\frac{1}{4}\times \frac{2}{4}\times \frac{4}{4}\times 4^{3}=8.$$

This would be an estimate of the neutral set size of *A* and constitutes the core idea behind the versatility calculations in [14,17,18]: the sequence constraints on each site are quantified and then multiplied to obtain the total number of sequences [14,17,18]. Previous work differs in how exactly these constraints are quantified and whether there are additional correction factors in the calculation (like [17]) and in this paper we build on the definitions in [14]: here, the constraintness of each site is quantified by a quantity called versatility, which varies continuously in a fixed range. If the versatility for site *i* is at its maximum, this means that the sequence *s* is equally likely to fold into structure *A*, regardless of the letter at site *i*, and therefore there are no constraints on site *i* [14], like site 3 in the example from equation (5.3). If the versatility for site *i* is at its minimum, this means that a sequence *s* can only fold into structure *A* if a specific letter is found at site *i* and so site *i* is fully constrained [14], like site 1 in our example. In between these extremes, like site 2 in our case, García-Martinín *et al.* [14] give a simple equation for how the versatility can be computed from the distribution of letters found at site *i* in the sequences folding into structure *A*, but since we are not working with sequence samples in this paper, this formula is not used.

In this paper, we make two adjustments to the method by García-Martín *et al.* [14]: we treat each base pair not as two separate sites, but instead as a single unit with an alphabet size of six (i.e. all allowed pairs: AU, UA, GC, CG, GU, UG) to simplify our calculations. Then a minimum-versatility paired site would be a site where a GC pair specifically is required for the structure to fold correctly (and even switching the order to give a CG pair is not permitted), whereas a maximum-versatility paired site would be a site where any valid base pair is sufficient. Considering a base pair as a single unit will also ensure that one key reason for the division of neutral sets into neutral components, as shown in [54], no longer exists and that we can therefore apply a versatility framework without worrying about the distinction between neutral components and neutral sets.

In order to avoid confusion when dealing with different alphabet sizes for paired and unpaired sites, we normalize all versatilities by dividing by the corresponding alphabet sizes (so versatilities are between zero and one). In addition, we assume that there are no constraints on loop sites except at the end of stacks since we have already accounted for loop free energies. With these adjustments, the equation for neutral set sizes from [14], which we introduced conceptually in equation (5.3), becomes

5.4 $${\tilde{N}}_{A}(G\leq x)=N_{c}\times v_{\mathrm{eos}}^{n_{\mathrm{eos}}}\times v_{\mathrm{bp}}^{n_{\mathrm{bp}}}.$$

Here *N*_*c*_ is the number of compatible sequences as defined in equation (2.1), *v*_eos_ the versatility of end-of-stack sites, *n*_eos_ the number of end-of-stack sites, *v*_bp_ the versatility of base pairs and *n*_bp_ the number of base pairs. In our calculations, all these quantities depend on the structure *A* and its free-energy terms. *N*_*c*_ is included here, but not in the original equation in [14] due to the different normalization of versatility values.

#### 5.6.2. Application of versatility treatment

To estimate the number of sequences for which equation (5.2) is satisfied, we make the following argument: the free energy contribution of sequence-dependent terms *G*_seq-dependent_ is primarily due to stacking terms and these tend to be most stabilizing if the relevant base pairs are GC base pairs, as seen in the parameter table in [2]. In the language of sequence constraints, as reviewed in §5.6.1, this argument can be rephrased as follows: if a stacking term is required to be highly stabilizing, the sequence at the relevant sites is constrained to GC/CG base pairs and these constraints mean that the versatility at these sites is low. We therefore use free-energy arguments to estimate the versatilities of the paired sites in a given structure and then use these versatilities to compute neutral set sizes, following equation (5.4). Then we apply the same concept to the end-of-stack sites. Our approach differs from the sample-based versatility models in [14,17], in that we do not derive the sequence constraints from large or even exhaustive sequence samples. Our calculations also differ from existing sample-free versatility approaches [14,55], which assume that the versatility of paired sites is a single constant value that applies to all structures. Our model, like sample-based approaches, accounts for the fact that different structures may have different sequence constraints at paired sites, but unlike sample-based approaches, these values are estimated directly from free-energy considerations for a specific structure and not inferred from a large sequence sample. In the following, we will discuss how we will use free-energy calculations to estimate the versatilities of paired sites and end-of-stack sites for a given structure.

#### 5.6.3. Versatility of paired sites

Since stacking free energies can have higher absolute values than end-of-stack terms in the Turner [2] model, we will assume that stacking terms are the primary sequence-dependent free energy terms. Then the free energy which individual stacking free energy contribution has to provide is *G*_stacking_ = *G*_max_/*n*_stack_.

Each stacking free energy contribution depends on the identity of two adjacent base pairs, as seen in the parameter table in [2]. These interdependencies between base pairs make exact calculations infeasible. However, to a first approximation we argue that the sequence in stacks is highly constrained if each stacking term has to contribute a high amount of stacking free energy, *G*_stacking_, as discussed above. We then make a simplifying assumption: we assume that the free energy contribution per stacking term is linearly related to the versatility in stacks. This linear relationship is modelled as a linear interpolation between *G*_stacking_ = −3.0 kcal mol^−1^ for GC/CG-only stacks (with a base pair versatility of *v*_*bp*_ = 2/6) and *G*_stacking_ = −0.5 kcal mol^−1^ for stacks with no sequence constraints (with a versatility of *v*_*bp*_ = 1). These values are all approximations to the full parameter table [2]: for example, only two values out of 36 parameters in the stacking free energy table are >−0.5 kcal mol^−1^ and thus this was chosen as an approximate upper limit.

The computed versatility values are only used for the low-energy calculation in equation (5.4) in this paper, but in the electronic supplementary material (section S4) the predicted base pair versatilities themselves are tested against base pair versatilities calculated from sequence samples in the low-energy map and good agreement is found.

#### 5.6.4. Versatility of end-of-stack sites

If the structure can be fully stabilized by the stacking interactions discussed above, we neglect the sequence dependence of end-of-stack terms (i.e. we assume that sites by the end of stacks can be occupied by any nucleotide and thus *v*_eos_ = 1). However, if even the most stabilizing stacking terms with *G*_stacking_ ≈ −3.0 kcal mol^−1^ are insufficient to satisfy equation (5.2), we perform an approximate calculation of additional free energy that could be provided if the sequence at end-of-stack terms is chosen carefully to stabilize the structure.

If the stacks are GC-only, we assume each end-of-stack term contributes an additional sequence-independent *a* = 0.2 kcal mol^−1^ since dangling ends and terminal mismatches are more stabilizing at the end of GC stacks, as seen in the parameter tables in [2]. If this is insufficient for the free energy criterion *G* ≤ *x*, additional constraints on end-of-stack terms are applied: we assume that each end-of-stack term can contribute up to an extra 0.9 kcal mol^−1^ in free energy if they are fully constrained (so that the maximum free energy contribution of end-of-stack terms is 1.6 kcal mol^−1^, corresponding to the most stabilizing terminal mismatch free energy in the Turner model [2]). Again, we use a linear interpolation between fully constrained sites (−0.9 kcal mol^−1^ and *v*_eos_ = 1/4 ) and fully versatile sites (0.0 kcal mol^−1^ and *v*_eos_ = 1).

If equation (5.2) cannot be fulfilled even after the most stabilizing choice for stacking and end-of-stack sites have been made, we assume that both stacking and end-of-stack sites have the smallest possible versatility values (*v*_*bp*_ = 2/6 and *v*_eos_ = 1/4) and that *N*(*G* ≤ *x*) is 0.1 times the value calculated from these versatilities, in order to account for additional sequence-dependent terms that are not considered in our approximate free energy calculations. Thus, we always assume that the input structure is designable (i.e. *N*_mfe_ > 0).

## Data Availability

The data behind this analysis and a Python implementation of the new method can be found at https://github.com/noramartin/free_energy_based_neutral_set_size_prediction. The data are provided in the electronic supplementary material [56].
